# The overall risk of malignancies is not increased in patients with Hirschsprung disease

**DOI:** 10.1007/s00383-020-04631-1

**Published:** 2020-02-26

**Authors:** Anna Löf Granström, Gabriella Cohn-Cedermark, Tomas Wester

**Affiliations:** 1 Department of Women’s and Children’s Health, Karolinska Institutet, Stockholm, Sweden; 2 Department of Oncology-Pathology, Karolinska Institutet and Karolinska University Hospital, Stockholm, Sweden; 3 Division of Pediatric Surgery, Astrid Lindgren Children’s Hospital, S3:02, Karolinska University Hospital, Solna, 17176 Stockholm, Sweden

**Keywords:** Hirschsprung disease, Cancer, Malignancy, Epidemiology

## Abstract

**Purpose:**

Hirschsprung disease (HSCR) has previously been associated with increased risk of medullary thyroid cancer. The aim of this study was to assess the overall risk of malignancies in patients with Hirschsprung disease in a population-based cohort.

**Methods:**

This was a nationwide, population-based cohort study. The study exposure was HSCR and the study outcome was malignancy. The cohort included all individuals with HSCR registered in the Swedish National Patient Register between 1964 and 2013 and ten age- and sex-matched controls per patient, randomly selected from the Population Register. Data were linked with the Swedish National Cancer Register to identify individuals with malignancy diagnosis.

**Results:**

The cohort comprised 739 individuals with HSCR (565 male) and 7390 controls (5650 male). Median age of the cohort was 19 years (range 2–49). In total nine (1.2%) individuals in the exposed cohort were diagnosed with malignancies compared to 57 (0.8%) in the non-exposed cohort (*p* = 0.195). Median age at malignancy diagnosis was 3 years (range 0–46) in the exposed group, compared to 23 (range 0–42), *p* = 0.132. No cases with medullary carcinoma of the thyroid were found in this cohort.

**Conclusions:**

There was no significant difference in risk of malignancies in the exposed group compared to the unexposed group.

## Introduction

Hirschsprung disease (HSCR) is a developmental defect of the enteric nervous system in the hindgut. The birth prevalence is 1 in 5000 living newborns [1]. The etiology is still unknown but HSCR is a multifactorial disease, probably caused by both environmental and genetic factors. HSCR can also be a part of a syndrome, most commonly trisomy 21 (Down syndrome).

The majority of HSCR mutations are identified in the *RET* gene; 50% of familial and 15–20% of sporadic cases of HSCR [2]. The *RET* proto-oncogene encodes a transmembrane receptor with tyrosine kinase activity. Germline mutations in *RET* are responsible for several inherited diseases as multiple endocrine neoplasia type 2 (MEN2) which is an autosomal‐dominant disorder. MEN2 *RET* gene mutations are mainly heterozygous, missense sequence changes found in *RET* exons 10, 11, and 13–16 [3]. Multiple endocrine neoplasia type 2 (MEN 2) includes the following phenotypes: MEN 2A, familial medullary thyroid carcinoma (FMTC), and MEN 2B. All three phenotypes involve high risk for development of medullary carcinoma of the thyroid (MTC) [4]. A Finnish study have shown an increased risk for MTC in patients with HSCR [5]. Individuals with trisomy 21 have been shown to have an increased risk for acute leukemia, testicular cancer as well as liver cancer [6, 7].

The aim of this study was to assess the overall risk for malignancies among Swedish patients diagnosed with Hirschsprung disease and to compare the risk with an age- and gender matched cohort.

## Methods

### Study design and settings

This was a nationwide, population-based cohort study during the observational period 1st of January 1964 to 31st of December 2013. The study exposure was HSCR and the primary study outcome was malignancy. Exposure and outcomes were assessed through linkage between the Swedish National Patient Register and the Swedish National Cancer Register.

### Data resources/registers

The Swedish National Patient Register was initiated in 1964 and contains prospectively collected information from all hospital admissions in Sweden since 1987. The register is maintained by the Swedish National Board of Health and Welfare and includes data on gender, age, geographical data, surgical procedures, date of admission and discharge, and primary and secondary diagnosis. The International Classification of Diseases (ICD) is used to register diagnosis. This classification has been modified over the years: ICD-7 in 1964–1968, ICD-8 in 1969–1986, ICD-9 in 1987–1996, and ICD-10 since 1997. The most recent validation of the register showed that the diagnoses are valid in 85–95% of the cases [8].

The Swedish Cancer Register is also maintained by the Swedish National Board of Health and Welfare. The register was initiated in 1958 and contains information about all malignancy diagnoses among Swedish citizens since then. Data as type of tumor according to ICD-classification, localisation, date of diagnosis, and age at diagnosis are recorded in the register.

The possibility to link these national registers is based on that all residents in Sweden get a unique ten-digit personal identification number after birth or immigration.

### Participants

The cohort was collected from the Swedish National Patient Register and Statistics Sweden. Data on the exposure, HSCR, was collected from the Swedish National Patient Register (ICD-7: 75,631, ICD-8: 751.39, ICD-9: 751D, ICD-10: Q431) during the study period. A total of 1267 individuals with these ICD codes were found. To confirm that they had HSCR and were not misdiagnosed by mistake, each case had to fulfill one of the following inclusion criteria:

1. HSCR as main diagnosis and a surgical intervention number specific for HSCR.
2. Admission to a pediatric surgical center at least twice, with a hospital stay of at least 4 days, at least once, and HSCR as main diagnosis for both hospital stays.
3. One long admission (≥ 4 days) at a pediatric surgical center once and more than one outpatient visit at a pediatric surgical center with HSCR as main diagnosis.

For instance, we wanted to avoid including neonates with suspected HSCR admitted for rectal suction biopsies where the biopsies turned out to be negative or patients admitted only to a hospital without pediatric surgery.

**Fig. 1:**
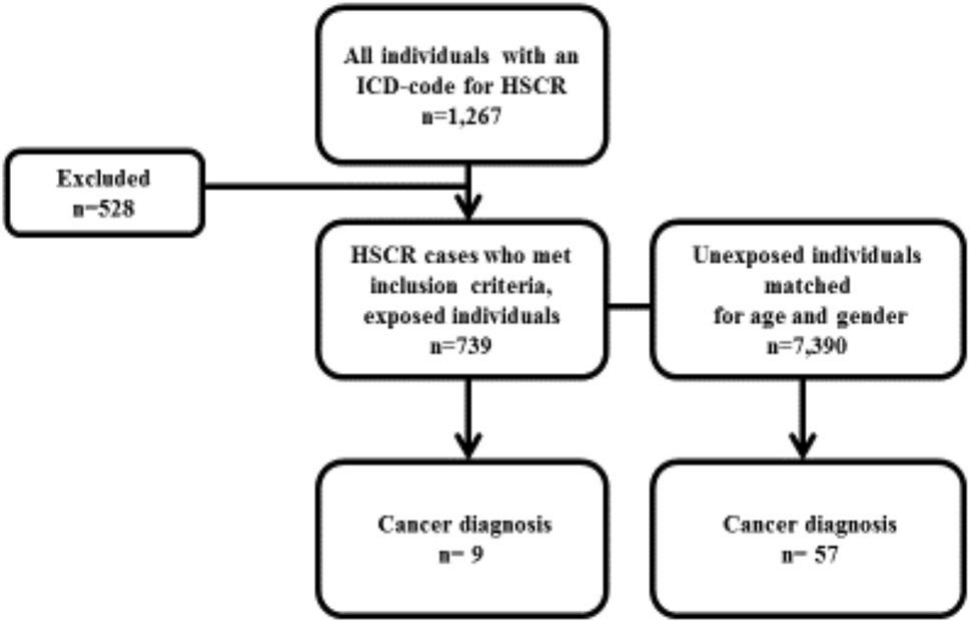
Study flowchart

Using these criteria, 528 individuals were excluded, ending up with 739 exposed cases. The unexposed individuals in the cohort were collected from the Swedish National Population Register and comprised ten unexposed individuals for each exposed individual matched for birth year and gender (*n* = 7390) (Fig. 1).

### Variables

The study outcome cancer diagnosis was defined as any registration of cancer in the Swedish National Cancer Register. HSCR is associated with trisomy 21, which was considered a potential bias. Individuals with Down syndrome were identified in both cohorts in the Swedish National Patient Register (ICD8: 759.3, ICD9: 758A, ICD10: Q90.0–90.9).

### Statistical analysis

The association between exposed and unexposed individuals was analyzed with R program [9]. Categorical data are presented as frequencies or proportions and analyzed with two-tailed Fisher’s exact test. Numerical data are presented as median and range and two-sided Mann–Whitney *U* test was used for analysis. *p* < 0.05 was considered statistically significant. A Cox regression analysis, presented as hazard ratio (HR) and 95% confidence interval (CI), was used for calculation of risk of cancer (Table 1).

**Table 1:** Differences in cancer diagnosis, age, and gender between the HSCR cohort and controls

	HSCR cohort *n* = 739	Control cohort *n* = 739	*p* value
Gender	565 males	5650 males	NS
Age at follow-up median (range)	19 (2–49)	19 (2–49)	NS
Individuals with cancer diagnosis (%)	9 (1.2)	57 (0.8)	NS
Male individuals with cancer diagnosis (%)	6 (1.1)	25 (0.4)	NS
Individuals with cancer diagnosis (%), trisomy 21 excluded	8 (1.1)	57 (0.8)	NS

### Ethics

The Regional Ethics Review Board in Stockholm approved the study.

## Results

The cohort comprised 739 individuals with HSCR (565 male) and 7390 controls (5650 male). Median age of the cohort was 19 years (2–49). Nine (1.2%) individuals with HSCR had received a malignancy diagnosis compared to 57 (0.8%) controls, *p* = 0.195. There was no significant difference in age at diagnosis between the exposed individuals whose median age was 3 years (0–46) compared to the unexposed individuals whose median age was 23 years (0–42), *p* = 0.132. The HR for receiving a cancer diagnosis among individuals with HSCR compared to the controls was 1.62 (CI 95% 0.8–3.28). Among the individuals with HSCR, 65 also had trisomy 21 compared to one person in the control group. When excluding all individuals with trisomy 21, eight (1.1%) individuals in the exposed group had a cancer diagnosis at the median age of 10.5 years (0–46) compared to 57 (0.8%) in the non-exposed group at the median age of 23 years (0–42), *p* = 0.255. The HR for receiving a cancer diagnosis among individuals with HSCR compared to the controls with trisomy 21 excluded was 1.6 (CI 95% 0.76–3.34). There was no significant difference when subgrouping for gender, HR among males 2.25 (CI 95% 0.86–5.89) and among females 1.09 (CI 95% 0.33–3.55).

The types of malignancy are summarized in Table 2. Two individuals with HSCR had malignancies classified to the central nervous system. One had a paraganglioma and the other suffered from a ganglioneuroblastoma. None of these individuals had a congenital central hypoventilation syndrome.

**Table 2:** Types of malignancy diagnosis in the HSCR cohort and controls

Type of malignancy diagnosis	HSCR cohort *N* = 9 (%)	Control cohort *N* = 57 (%)
Hematological	3 (33.3)	7 (12.3)
Nervous system	2 (22.2)	8 (14)
Skin	1 (11.1)	3 (5.3)
Gastrointestinal	1 (11.1)	4 (7)
Gynecological	1 (11.1)	20 (35.1)
Testicular	1 (11.1)	6 (10.5)
Ear, nose, throat	–	2 (3.5)
Breast	–	2 (3.5)
Skeletal	–	2 (3.5)
Connective tissue	–	2 (3.5)
Thyroid	–	1 (1.8)

## Discussion

### Key results

This is a large national population-based register cohort study showing no increased risk of malignancy among individuals with HSCR compared to healthy controls. There was no significant difference in age at diagnosis or in gender. There were no HSCR cases diagnosed with thyroid cancer during the study period.

### Limitations

This study was based on prospectively collected national register data, previously shown to have high validity. To reduce the risk for misclassification, specific inclusion criteria were defined in advance to identify the exposure of HSCR. Since this is a register-based study, no histopathology reports were available for HSCR diagnosis. This is a limitation of the study, since we may have included patients without HSCR, but also excluded patients with HSCR.

To be able to study the malignancy risk, controls were randomly selected from Statistics Sweden, reducing the risk for selection bias. To decrease the risk for confounders, the controls were matched for birth year and gender. One other confounder is the fact that HSCR is associated with Down syndrome. Individuals with trisomy 21 are shown to have an increased risk for acute leukemia, testicular cancer as well as liver cancer [6, 7]. There was one individual with HSCR and trisomy 21 who also was diagnosed with acute leukemia. We analyzed data adjusted for trisomy 21 and could not show any effect on the cancer rate.

The study included cases with HSCR since the beginning of the Swedish Patient Register in 1964 and the controls. Although some of the malignancies in this study as testicular cancer and medullary carcinoma of the thyroid is most common between individuals aged 20–40 years, malignancies become more common with age. This may cause a limitation to this study since we cannot study the risk for malignancies in individuals at older age than 49 years of age. From the available data, we can study the risk for malignancies at a younger age, but unfortunately, we can’t study risk at an older age yet.

### Interpretation

In the literature, there are just a few papers on HSCR and malignancies in general. There are a few papers on HSCR and MTC. Pakarinen et al., found seven cases with malignancies with a standardized incidence ratio of 3.5 (95% CI 1.4–7.3). Two male patients at the ages of 34 and 37 developed MTC [5]. The same group have also published a study on thyroid cancer and *RET* gene mutations showing that both individuals with MTC had a *RET* gene mutation in exon 10. They also found one individual with papillary thyroid tumor without *RET* gene mutation [10]. In a study of 60 HSCR patients, screened for *RET* gene mutations, MEN 2 *RET* gene mutations were found in three individuals. They declared that it is difficult to predict tumor risk for patients with familial or sporadic Hirschsprung disease and that in combined MEN2A/Hirschsprung disease families *RET* gene testing, tumor screening, and prophylactic thyroidectomy are indicated as in MEN2A [11].

In our study, we found nine HSCR individuals with malignancy, categorized to hematological malignancies, CNS tumors, skin tumor, cervix tumor, testicular tumor and one individual with a tumor in the small intestines. We found no thyroid malignancies in our study cohort.

The finding of one paraganglioma and one ganglioneuroblastoma is of interest since these tumors are derived from the neural crest.

### Generalizability

Being based on a national population-based study, these results are considered highly generalizable. Individuals with HSCR do not have an increased risk of cancer diagnosis at an early age compared to an unexposed cohort.
